# Brain activation differences in schizophrenia during context-dependent processing of saccade tasks

**DOI:** 10.1186/s12993-016-0103-2

**Published:** 2016-06-24

**Authors:** A. L. Rodrigue, B. P. Austin, K. A. Dyckman, J. E. McDowell

**Affiliations:** Department of Psychology, University of Georgia, Psychology Building 125 Baldwin Street, Athens, GA 30602 USA; Department of Medicine, School of Medicine and Public Health, University of Wisconsin, 2500 Overlook Terrace, Madison, WI 53705 USA

**Keywords:** Schizophrenia, Context, fMRI, Antisaccades, Prosaccades

## Abstract

**Background:**

Brain function in schizophrenia has been probed using saccade paradigms and functional magnetic resonance imaging, but little information exists about how changing task context impacts saccade related brain activation and behavioral performance. We recruited schizophrenia and comparison subjects to perform saccade tasks in differing contexts: (1) two single task runs (anti- or pro-saccades alternating with fixation) and (2) one dual task run (antisaccades alternating with prosaccades).

**Results:**

Context-dependent differences in saccade circuitry were evaluated using ROI analyses. Distinction between anti- and pro-saccade activation across contexts (single versus dual task) suggests that the schizophrenia group did not respond to context in the same way as the comparison group.

**Conclusions:**

Further investigation of context processing effects on brain activation and saccade performance measures informs models of cognitive deficits in the disorder and enhances understanding of antisaccades as a potential endophenotype for schizophrenia.

**Electronic supplementary material:**

The online version of this article (doi:10.1186/s12993-016-0103-2) contains supplementary material, which is available to authorized users.

## Background

Context processing is defined as the ability to recognize and maintain information necessary for the execution of task-relevant responses [1]. Context manipulations can range in specificity from using previously encountered stimuli within a task, to more global instances of using task instructions to bias responses and guide behavior [2]. People with schizophrenia show impairments in context processing [2–5]. These deficits are apparent when tasks involve suppression of prepotent responses in reference to contextual cues and are related to deficits in the functioning of prefrontal cortex (PFC) and its circuitry [1, 4, 6–10].

Saccade tasks are useful paradigms for studying context processing deficits in schizophrenia. Saccades are fast eye movements that redirect gaze and require either a stimulus-driven glance toward (prosaccade), or a controlled glance away (antisaccade), from a suddenly appearing peripheral stimulus. Studies using functional magnetic resonance imaging (fMRI) have shown that saccade circuitry includes a number of cortical and sub-cortical regions, although antisaccades typically require greater activation in existing circuitry, as well as recruitment of additional regions, such as PFC [11–14]. People with schizophrenia show relatively preserved prosaccade performance and poor antisaccade performance, evidenced by higher error rates, slower correct response times [15–17] and under-activation of associated control regions, including PFC [12, 18, 19]. Furthermore, deficits in antisaccade performance and associated brain activation may be considered endophenotypes for schizophrenia [20, 21], making investigation of context-dependent effects on saccade performance measures and brain activation an important area of study.

One means of manipulating context in saccade paradigms is via the presentation of single and dual task runs. A single task run consists of one saccade type (blocks of anti- or pro-saccades alternating with blocks of fixation, e.g. AS-Fix-AS-Fix), whereas a dual task run consists of two saccade types (blocks of antisaccades alternating with blocks of prosaccades, e.g. AS-PS-AS-PS). This type of context processing is reflective of more global processes, where task instructions are used to bias certain behavioral responses. Results of Dyckman et al. [22] demonstrate that when anti- and pro-saccades are performed in single vs. dual task runs in healthy people, there are quantifiable differences in saccade circuitry activation. Regions that show greater anti- than pro-saccade activation in the single task runs, do not always show the same pattern in the dual task run. Context processing deficits associated with saccade tasks in schizophrenia have been reported in studies using electroencephalography (EEG) and magnetoencephalography (MEG). When anti- and pro-saccade trials are performed in the same run, people with schizophrenia show smaller neural differentiation between cues that signal different trial types [23–25]. These studies, however, only evaluate differences between anti- and pro-saccades when they are performed in the same run and/or focus on a more specific type of context processing: cue responses to individual trials. This differs from the more global evaluation of context in Dyckman et al. [22].

The primary goal of this study is to quantify context processing of saccade tasks in schizophrenia by comparing behavioral performance and associated brain activation in single saccade task runs versus a dual saccade task run using fMRI (similar to Dyckman et al. [22]). To document context-dependent differences in saccade circuitry, we evaluate the blood oxygen level dependent (BOLD) signal using region of interest (ROI) analyses. We hypothesize that people with schizophrenia will fail to show differentiation in saccade circuitry activation based on contextual information.

## Methods

### Subjects

We recruited thirty DSM-IV-TR diagnosed schizophrenia subjects (age M = 37.8 years, SD = 10.5; 60 % male, 27 right handed) [26] and twenty-nine comparison subjects (age M = 36.3 years, SD = 11.2; 55 % male, 26 right handed). Comparison subjects were given the non-patient edition of the structured clinical interview for DSM-IV-TR [27] and the schizotypal personality questionnaire (SPQ) [28] to rule out existing psychopathology. All subjects signed an informed consent and were screened for confounding factors: head trauma, drug use, and/or criteria related to MRI compatibility. This study was approved by the University of Georgia (UGA) Institutional Review Board.

### Procedure

Imaging was performed at the UGA Bio-Imaging Research Center with a GE Excite HD 3.0T MRI scanner (Milwaukee, Wisconsin, USA). Subjects were given task-specific instructions before being positioned in the scanner. Heads were stabilized with foam padding and a forehead strap. Subjects viewed stimuli through a dual mirror box (16 cm above and in front of the eyes) on a screen at their feet (174 cm from the nasion).

**Fig. 1:**
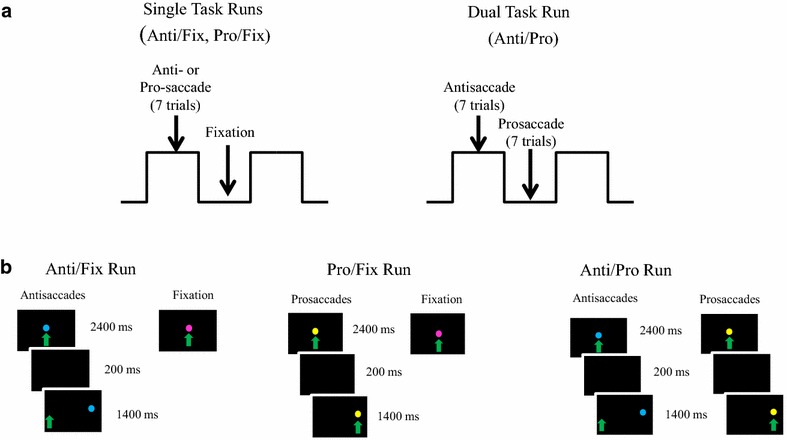
Imaging design and stimuli. **a** Imaging design. Three block designed runs: two single task runs and a dual task run. There were 13 blocks in each run, 7 fixation blocks and 6 antisaccade blocks: each block lasted 28 s for a total run time of 6 min and 4 s. **b** Stimuli and stimuli timing. All trials during saccade tasks had the same timing: 2400 ms fixation stimulus, 200 ms gap, and 1400 ms peripheral stimulus. *Stimuli color* was used to cue task instructions. Saccades were made ±5º or 10º in the *horizontal plane*. *Green arrows* indicate correct direction of gaze and were not visible to the subject

Imaging acquisition included a fast 3D T1-weighted structural scan BRAVO protocol (TE = 4.6 ms, TR = 10.8 ms, flip angle = 13°, matrix = 352 × 224, FOV = 24 cm, in-slice resolution .68 × 1.07, 1.2 mm slice thickness, 150 slices, scan time 3 min 7 s) to determine the angle of acquisition along the AC-PC line. An additional T1-weighted 3D FSPGR sequence (TE = Min-Full, TR = 7.8 ms, flip angle = 20°, matrix = 256 × 256, FOV = 24 cm, in-slice resolution .9375 × .9375, 1.2 mm slice thickness, 150 axial slices, scan time 6 min 20 s) was acquired to obtain a high resolution image of subjects’ brain anatomy. Three T2*-weighted gradient echo whole brain EPI scans also were collected (TE = 30 ms, TR = 2000 ms, flip angle = 90°, matrix = 64 × 64, FOV = 22 cm, in-slice resolution 3.4375 × 3.4375, 4 mm slice thickness, 33 slices, oblique acquisition (AC-PC aligned), 180 volumes, scan time 6 min 12 s) while eye movements were recorded (MEyeTrack LR, SensoMotoric Instruments, Inc., Berlin, Germany). During the three T2* scans, subjects performed two single task runs of saccades (blocks of anti- or pro-saccades alternating with blocks of fixation-Anti/Fix and Pro/Fix respectively) and one dual task run of saccades (blocks of antisaccades alternating with blocks of prosaccades-Anti/Pro). Saccade blocks in each run contained 7 trials. The Anti/Fix and Pro/Fix runs, therefore, contained 42 trials each (6 blocks × 7 trials) and the Anti/Pro run contained 42 antisaccade trials (6 blocks × 7 trials) and 47 prosaccade trials (7 blocks × 7 trials). Run order was counterbalanced across subjects. See Dyckman et al. [22] for saccade task instructions. Task parameters, stimuli, and timing are shown in Fig. 1.

### Analysis

#### Behavior

Eye movement data were scored in MATLAB (The Mathworks Inc., Natick, MA, USA). Saccade performance measures included percent correct (PC) [(number of correct trials/total number of usable trials) ×100] and correct reaction time (RT) (time in ms between peripheral stimulus presentation and saccade start) for each subject and each saccade task in the three imaging runs (Anti/Fix, Pro/Fix, Anti/Pro). Percent of corrected errors [(number of corrected errors/total number of errors) ×100] for antisaccades also was calculated in order to assess subjects’ understanding of the task. Eye movements for 2/30 schizophrenia and 2/29 comparison subjects were not obtained due to technical difficulties. Subjects with missing eye movement data sufficiently performed all tasks and were included in the imaging analyses.

#### fMRI

Preprocessing of functional images was completed with analysis of functional neuroimages (AFNI) [29] software and included despiking, slice timing correction, registration to a representative volume for movement, alignment of functional data to anatomy, smoothing with a 4 mm full-width at half-maximum (FWHM) Gaussian filter, and scaling each voxel to a mean of 100. Functional images for each subject were masked using the subject’s anatomical image and warped to talairach space in preparation for reference function estimation via a probabilistic independent component analysis (PICA) (using MELODIC [30] in FMRIB Software Library [31]). Blocked design fMRI data is commonly analyzed by testing voxel time courses against hypothesized reference functions created by convolving block onsets and their durations with the hemodynamic response. These reference functions do not fully characterize the data, however, and are likely unrealistic when modeling data with multiple contributing sources of signal and noise [32]. We used PICA to obtain a data-driven reference function for use in a later GLM analysis, similar to that suggested in McKeown et al. [32]. PICA was chosen for reference function estimation because of its agnostic approach and its ability to reduce problems of overfitting, which can be the case when using hypothesis driven reference functions and GLM methods [30]. Furthermore, analyses in the present study were meant to closely match those in Dyckman et al. [22] to ensure comparability of results. Each run was averaged across subjects followed by concatenation of the three runs in space. Averaging across subjects is one alternative when estimating component maps using ICA. With a large number of subjects, it reduces the computational load, yet accurately estimates associated time courses [33]. PICA returned 43 spatially independent components. By referring to a scree plot of the percent of variance accounted for and visual inspection of the time course for each component, we selected the first four as ideal reference waveforms. Components are ordered by their explained variance and ranged from 7.91 to 1.6 %; the percentage associated with the 4th component was 3.5 %. The first four components showed the same peak frequency and time course as the experimental design. A GLM (using AFNI’s 3dFIM+) was then performed for each subject between the time series of the first four PICA components and the BOLD time series in each voxel for each run. Movement estimates from registration in the preprocessing stage and functions characterizing scanner drift (linear, quadratic, and cubic) were used as regressors of no interest. For each run, results returned a voxel-wise best fit correlation co-efficient with one of the four task related components (see Dyckman et al. [22]). From the chosen component, percent signal change was calculated for each voxel. This value was used to characterize activation for each run: activation related to antisaccades compared to fixation in the Anti/Fix run, activation related to prosaccades compared to fixation in the Pro/Fix run, and activation related to antisaccades compared to prosaccades in the Anti/Pro run.

Activation in saccade circuitry was evaluated with ROI analyses. ROIs were 8 mm spheres centered on coordinates reported in Dyckman et al. [22] and included supplementary eye fields (SEF), lateral frontal eye fields (latFEF), medial frontal eye fields (medFEF), prefrontal cortex (PFC), precuneus, cuneus, middle occipital gyrus (MOG), inferior parietal lobule (IPL), striatum, and thalamus. For each individual and for each run, ROI spheres were overlaid on functional maps. The percent signal change from voxels encompassed by the ROI masks were averaged and output as a single value for each ROI. The average percent signal change across subjects was then calculated for each ROI in each run. Differences between anti- and pro-saccade activation in the single task runs (Anti/Fix vs. Pro/Fix) were quantified with dependent sample t-tests. Differences between anti- and pro-saccade activation in the dual task run were quantified with one sample t-tests (Anti/Pro vs. 0).

The effect of context (difference in activation between anti- and pro-saccades in the single vs. dual task run) was evaluated with a subtraction method. Activation in the Pro/Fix run was subtracted from that in the Anti/Fix run for all ROIs in each subject. This difference was compared to activation in the Anti/Pro run (because of the block design nature, the Anti/Pro run was already the difference in activation between anti- and pro-saccades). To quantify significant differences between anti- and pro-saccade activation in the single vs. dual task runs, dependent *t* test were done within each group. All tests were two-tailed and thresholds for significance were set at p = .05.

## Results

### Behavior

Results are summarized in Table 1.

**Table 1:** Behavioral results summary

	Percent correct				Reaction time (ms)			
	C (n = 27)	SZ (n = 28)	T-statistic, p value	Cohen’s d	C (n = 27)	SZ (n = 27)	T-statistic, p value	Cohen’s d
*Antisaccades*								
Anti/Fix	71.0 (25)	53.8 (30)	t(53) = 2.2, .03*	.60	287 (81)	303 (61)	t(50) = −.82, .42	−.23
Anti/Pro	76.0 (24)	55.5 (31)	t(53) = 2.7, .01*	.74	279 (68)	313 (89)	t(50) = −1.5, .13	−.42
*Prosaccades*								
Pro/Fix	98.8 (1.0)	98.7 (2.1)	t(53) = .20, .84	.05	175 (23)	178 (31)	t(52) = −.31, .76	−.09
Anti/Pro	97.5 (4.0)	95.0 (13)	t(53) = .95, .35	.26	180 (26)	181 (34)	t(52) = −.05, .96	−.01

Saccade performance measures showing percent correct and correct reaction time (mean (SD)) by saccade type (anti- or pro-saccade) in each of the three runs (Anti/Fix, Pro/Fix, and Anti/Pro). T-statistics and corresponding p values are for comparisons between C and SZ groups. The SZ group made significantly more antisaccade errors in each run. There were no significant differences across single and dual task runs for either group

*C* comparison group, *SZ* schizophrenia group

* p < .05

The schizophrenia group generated significantly more antisaccade errors than the comparison group in both the single and dual task runs (see Table 1 for test statistics). Saccade performance measures did not significantly differ between the single and dual task runs in the schizophrenia group (Antisaccades: PC [t(27) = −.48, p = .64], RT [t(25) = −.84, p = .41]; Prosaccades: PC [t(27) = 1.48, p = .15], RT [t(26) = −.46, p = .65]) or in the comparison group (Antisaccades: PC [t(26) = −1.64, p = .11], RT [t(25) = .68, p = .50]; Prosaccades: PC [t(26) = 1.89, p = .07], RT [t(26) = −1.31, p = .19]). Percentage of corrected antisaccade errors was above eighty percent (SZ: M = 84 %, SD = 25; C: M = 90 %, SD = 16) and did not significantly differ between the two groups [t(52) = 1.08, p = .28].

### fMRI

#### Overall results

Both the schizophrenia and comparison groups showed greater anti- than pro-saccade activation in a majority of ROIs, regardless of whether they were performed in the single or dual task run, although there were two exceptions. First, in both groups, the MOG showed more pro- than anti-saccade activation in the single and dual task runs. Second, in the comparison group, the IPL showed more anti- than pro-saccade activation in the dual task run only.

#### Context results

**Fig. 2:**
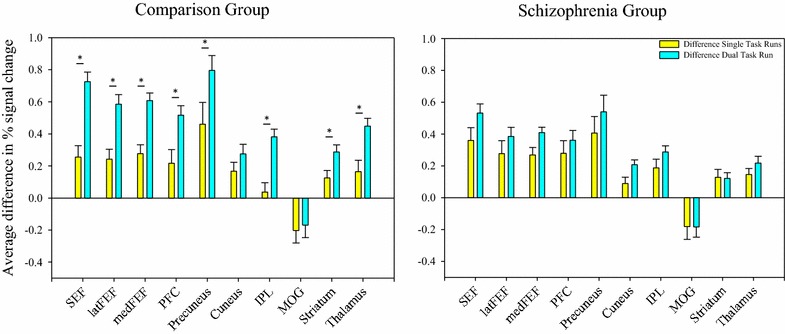
Context results. *Bars* show average difference in percent signal change (difference in activation and SE) for each ROI. *Yellow bars* show the difference between anti- and pro-saccade activation when performed in the single task runs (Anti/Fix minus Pro/Fix). *Blue bars* show the difference between anti- and pro-saccade activation when performed in the dual task run (Anti/Pro). Positive values in each indicate anti- > pro-saccade activation, negative values indicate pro- > anti-saccade activation. *p < .05

Subjects performed saccades in two contexts: single task runs and a dual task run. Context was evaluated by comparing the difference between anti- and pro-saccade activation in the single task runs (Fix/Anti-Fix/Pro) to that in the dual task run (Anti/Pro). The comparison group exhibited a consistent and robust pattern of activation that was context-dependent across a majority of the a priori defined ROIs in that the difference between anti- and pro-saccade activation was greater in the dual task run than in the single task runs. The schizophrenia group did not exhibit such a pattern. The difference between anti- and pro-saccade activation was similar regardless of context for all ROIs (Fig. 2).

## Discussion

Context processing deficits in schizophrenia are evidenced by poor behavioral performance and disrupted brain activation patterns. Saccade tasks are reliably used to probe brain function in schizophrenia and provide a unique means by which to look at context processing deficits in this disorder. Subjects performed saccade tasks during fMRI in two contexts: single and dual task runs. Context-dependent modulation of saccade circuitry in schizophrenia and comparison groups was evaluated using ROI analyses.

The pattern of brain activation in the schizophrenia group was different than that demonstrated by the comparison group (Fig. 2). In the comparison group, activation was modulated by context. The dual task run was associated with greater antisaccade activation, resulting in larger differences between anti- and pro-saccade activation than in the single task runs. Antisaccades typically require greater circuitry activation than prosaccades [34], but contexts that impose a higher cognitive load, like the Anti/Pro run, require more neural resources and result in increased brain activation [35–38]. The schizophrenia group did not exhibit such a pattern, and instead showed differences between anti- and pro-saccade activation that were similar across contexts (Fig. 2). This similarity in the schizophrenia group could arise from increases in both anti- and pro-saccade activation in the dual task run (which would leave the difference between them unchanged). People with schizophrenia show similar levels of brain activity (as measured by MEG) as comparison subjects, however, for prosaccades when they are done in the same run with antisaccades [25]. It is more likely then that the schizophrenia group failed to increase activation during antisaccades like the comparison group in the dual task run. This is consistent with reports that people with schizophrenia show less circuitry activation than comparison subjects at higher cognitive loads [39–41]. More specific to our study design, Barbalet et al. [42] showed people with schizophrenia exhibit similar levels of brain activation in complex, dual-task runs as in simple, single-task runs. People with schizophrenia often have difficulties recruiting additional neural resources when the cognitive load or the contextual complexity increases [39, 41] and reach the limit of their ability to recruit those neural resources at lower thresholds than comparison subjects [43].

One important consideration related to the studies cited above is that all report behavioral deficits paired with neural under-activation in schizophrenia when going from a simple to a more complex context [40, 41], suggesting context was recognized, but not appropriately invoked to cope with differing task demands. In the current study, behavioral performance for anti- and pro-saccades in the schizophrenia group did not differ across contexts. It could be that brain differences are more sensitive to context manipulations than behavior. Because of the predictable nature of the trials in the Pro/Anti run, behavior may have been preserved in the face of differences in brain activation.

Lack of difference between anti- and pro-saccade activation in the dual task run in the schizophrenia group could be due to task switching deficits, although this does not seem to be the most likely explanation. Schizophrenia subjects showed similar switch costs as comparison subjects in both error rate and reaction time measures for single and dual task runs (see Additional file 1). Additionally, other studies using runs including anti-and pro-saccades have shown that task switching is preserved in schizophrenia [44–46]. Our study also utilized an fMRI blocked design. Block designs do not allow for the separation of individual trials and are sensitive to sustained activation to a train of stimuli [47]. Activation, therefore, is a result of averaging brain responses to many closely spaced trials and do not account for transient responses to switch trials that may start a task block. Our results could be due to deficits in set maintenance in schizophrenia, a related, but distinct process from task set switching involved in performing runs with two tasks [48]. Set maintenance refers to the ability to regulate how much competing task sets interfere with the present task set. Kieffaber and colleagues [49, 50] found that people with schizophrenia are impaired in their ability to sustain encoding processes related to the current response implementation. This is similar to other studies that have found intact task switching in schizophrenia, but problems in working memory for global task context [1, 51]. This lack of sustainability may underlie the lack of antisaccade activation in the schizophrenia group since task set maintenance results in sustained and tonic activation (which is more likely detectable with block designs) of brain regions that overlap with those in our study [52]. It is important to note that studies of set maintenance deficits in schizophrenia typically only use dual task paradigms. In this case, mixing cost effects, which are attributed to behavioral differences between single task and dual task runs, cannot be accounted for. In our study, mixing costs were not apparent (see Additional file 1; Table 1). Furthermore, both groups showed similar behavioral patterns across contexts even though the schizophrenia group displayed generalized performance deficits (significantly higher error rates and non-significantly slower RTs).

People with schizophrenia exhibited worse behavioral performance on the antisaccade task (in both contexts), which is consistent with other saccade studies [53]. It is possible that lack of greater activation in the Anti/Pro run in the schizophrenia group was due to fewer correct trials. The schizophrenia group, however, showed similar levels of activation as the comparison group in the Anti/Fix run, despite similar differences in antisaccade behavior. People with schizophrenia activated the same amount as comparison subjects in the Anti/Fix with less behavioral benefit, reflecting inefficient neural processing often seen in schizophrenia [54]. Differing behavioral performance between the two groups, therefore, does not seem to account for differences in Anti/Pro activation. Differences between the two groups could also be an effect of psychotropic medication although poor context processing and associated brain dysfunction is a persistent feature in schizophrenia regardless of medication status [4, 8] and furthermore, is not a general feature of psychopathology [4, 55].

This study partially replicated results from Dyckman et al. [22], which included healthy people performing similar single and dual task saccade runs. That study found neither prosaccade activation in the thalamus or PFC in the Pro/Fix run nor a difference between anti- and pro-saccade activation in the dual task run in some ROIs. It is possible that these discrepancies were due to a problem of underestimation in the PICA. Dyckman et al. [22] used three PICA components in the GLM analysis. The use of a fourth PICA component in the current study may have accounted for additional variation, resulting in greater measurable activation in the Anti/Pro run. It is also true that Dyckman et al. [22] used a lower field strength than the current study (1.5 vs. 3 T). Although the contributions of this variable may be small, there are reports that frontal, thalamic, striate, and extrastriate regions may be better detected with a higher field strength [56]. The participants in the previous study were also all female, college-age women, whereas our sample was community-based and had an average age of around thirty. Sample characteristics, including those related to age, education level, etc. may also account for study differences.

## Conclusions

Based on our imaging analyses, the schizophrenia group did not respond to context in the same way as the comparison group. This could have been due to concomitant increases in both anti- and pro-saccade activation or a failure to increase antisaccade activation alone in the schizophrenia group. We provide support for the latter, although future studies should include runs with comparable baselines so that both anti- and pro-saccade activation can be evaluated in both contexts.

Disrupted context processing of saccade tasks in schizophrenia could have important implications. Antisaccades are considered possible endophenotypes for the disorder and are commonly used to index cognitive control. Additionally, saccade tasks may inform research on context processing deficits in schizophrenia, which may contribute to a number of chronic disturbances in cognition and behavior that impact daily functioning.

## Additional file

10.1186/s12993-016-0103-2 Trial Position and Performance Costs. Graphs show mean performance measures (SE) for antisaccades (left) and prosaccades (right) in each context plotted by trial position within a block. The schizophrenia group did not differ from the comparison group in either switch costs or mixing costs (similar pattern of means between the solid line and hashed line within each group). These patterns make it unlikely that differential switch or mixing costs between the two groups are responsible for the reported group-wise neural differences. C = comparison group, SZ = schizophrenia group.

## Abbreviations

FMRI: functional magnetic resonance imaging
PFC: prefrontal cortex
AS: antisaccade
PS: prosaccade
EEG: electroencephalography
MEG: magnetoencephalography
BOLD: blood oxygen level dependent
ROI: region of ineterst
DSM-IV TR: Diagnotsic and Statistical Manual Four-Revised
M: mean
SD: standard deviation
SPQ: Schizotypal Personality Questionnaire
UGA: University of Georgia
MRI: magnetic resonance imaging
mm: millimeter
cm: centimeter
min: minute/s
s: second/s
ms: millisecond/s
GE: General Electric
BRAVO: BRAin VOlume imaging
FSPGR: Fast SPoiled Gradient Recalled Acquisition in the Steady State
AC-PC: anterior commissure-posterior commissure
FOV: field of view
EPI: echo-planar imaging
TR: repitition time
TE: echo time
PC: percent correct
RT: reaction time
FWHM: full-width half-maximum
PICA: probabilistic independent components analysis
MELODIC: Multivariate Exploratory Linear Optimized Decomposition into Independent Components
ICA: independent component analysis (ICA)
GLM: general linear model
AFNI: Analysis of Functional NeuroImages
SEF: supplementary eye fields
medFEF: medial frontal eye fields
latFEF: lateral frontal eye fields
MOG: middle occipital gyrus
IPL: inferior parietal lobule
C: comparison
SZ: schizophrenia
p: probability
SE: standard error

## References

[1] Cohen JD, Barch DM, Carter C, Servan-Schreiber D (1999). Context-processing deficits in schizophrenia: converging evidence from three theoretically motivated cognitive tasks. J Abnorm Psychol.

[2] Cohen JD, Servan-Schreiber D (1992). Context, cortex, and dopamine: a connectionist approach to behavior and biology in schizophrenia. Psychol Rev.

[3] Servan-Schreiber D, Cohen JD, Steingard S (1996). Schizophrenic deficits in the processing of context. A test of a theoretical model. Arch Gen Psychiatry.

[4] MacDonald AW, Carter CS, Kerns JG, Ursu S (2005). Specificity of Prefrontal Dysfunction and Context Processing Deficits to Schizophrenia in Never-Medicated Patients With First-Episode Psychosis. Am J Psychiatry.

[5] Miller EK (2000). The prefrontal cortex and cognitive control. Nat Rev Neurosci.

[6] Stratta P, Daneluzzo E, Bustini M, Prosperini P, Rossi A (2000). Processing of context information in schizophrenia: relation to clinical symptoms and WCST performance. Schizophr Res.

[7] Javitt DC, Shelley A-M, Silipo G, Lieberman JA (2000). Deficits in auditory and visual context-dependent processing in schizophrenia: defining the pattern. Arch Gen Psychiatry.

[8] Barch DM, Carter CS, MacDonald AW, Braver TS, Cohen JD (2003). Context-processing deficits in schizophrenia: diagnostic specificity, 4-week course, and relationships to clinical symptoms. J Abnorm Psychol.

[9] Breton F, Planté A, Legauffre C, Morel N, Adès J, Gorwood P, Ramoz N, Dubertret C (2011). The executive control of attention differentiates patients with schizophrenia, their first-degree relatives and healthy controls. Neuropsychologia.

[10] Henik A, Salo R (2004). Schizophrenia and the stroop effect. Behavioral and cognitive neuroscience reviews.

[11] McDowell JE, Dyckman KA, Austin BP, Clementz BA (2008). Neurophysiology and neuroanatomy of reflexive and volitional saccades: evidence from studies of humans. Brain Cogn.

[12] McDowell JE, Brown GG, Paulus M, Martinez A, Stewart SE, Dubowitz DJ, Braff DL (2002). Neural correlates of refixation saccades and antisaccades in normal and schizophrenia subjects. Biol Psychiatry.

[13] Sweeney J, Mintun M, Kwee S, Wiseman M, Brown D, Rosenberg D, Carl J (1996). Positron emission tomography study of voluntary saccadic eye movements and spatial working memory. J Neurophysiol.

[14] Rosano C, Krisky CM, Welling JS, Eddy WF, Luna B, Thulborn KR, Sweeney JA (2002). Pursuit and saccadic eye movement subregions in human frontal eye field: a high-resolution fMRI investigation. Cereb Cortex.

[15] Ettinger U, Kumari V, Crawford TJ, Corr PJ, Das M, Zachariah E, Hughes C, Sumich AL, Rabe-Hesketh S, Sharma T (2004). Smooth pursuit and antisaccade eye movements in siblings discordant for schizophrenia. J Psychiatr Res.

[16] Ettinger U, Picchioni M, Hall M-H, Schulze K (2006). Antisaccade performance in monozygotic twins discordant for schizophrenia: the maudsley twin study. Am J Psychiatry.

[17] McDowell JE, Myles-Worsley M, Coon H, Byerley W, Clementz BA (1999). Measuring liability for schizophrenia using optimized antisaccade stimulus parameters. Psychophysiology.

[18] Camchong J, Dyckman KA, Austin BP, Clementz BA, McDowell JE (2008). Common neural circuitry supporting volitional saccades and its disruption in schizophrenia patients and relatives. Biol Psychiatry.

[19] Tu PC, Yang TH, Kuo WJ, Hsieh JC, Su TP (2006). Neural correlates of antisaccade deficits in schizophrenia, an fMRI study. J Psychiatr Res.

[20] Calkins ME, Iacono WG, Ones DS (2008). Eye movement dysfunction in first-degree relatives of patients with schizophrenia: a meta-analytic evaluation of candidate endophenotypes. Brain Cogn.

[21] McDowell JE, Myles-Worsley M, Coon H, Byerley W, Clementz BA (1999). Measuring liability for schizophrenia using optimized antisaccade stimulus parameters. Psychophysiology.

[22] Dyckman KA, Camchong J, Clementz BA, McDowell JE (2007). An effect of context on saccade-related behavior and brain activity. NeuroImage.

[23] Reuter B, Herzog E, Endrass T, Kathmann N (2006). Brain potentials indicate poor preparation for action in schizophrenia. Psychophysiology.

[24] Klein C, Heinks T, Andresen B, Berg P, Moritz S (2000). Impaired modulation of the saccadic contingent negative variation preceding antisaccades in schizophrenia. Biol Psychiatry.

[25] Manoach DS, Lee AK, Hämäläinen MS, Dyckman KA, Friedman JS, Vangel M, Goff DC, Barton JJ (2013). Anomalous use of context during task preparation in schizophrenia: a magnetoencephalography study. Biol Psychiatry..

[26] First MB, Spitzer RL, Gibbon Miriam and Williams, Janet BW. Structured clinical interview for DSM-IV-TR axis I disorders, research version, patient edition. In: (SCID-I/P). New York: Biometrics Research, New York State Psychiatric Institute; 2002.

[27] First MB, Spitzer RL, Gibbon Miriam and Williams, Janet BW. Structured clinical interview for DSM-IV-TR axis i disorders, research version, non-patient edition. In: (SCID-I/NP). New York: Biometrics Research, New York State Psychiatric Institute; 2002.

[28] Raine A (1991). The SPQ: a scale for the assessment of schizotypal personality based on DSM-III-R criteria. Schizophr Bull.

[29] Cox RW (1996). AFNI: software for analysis and visualization of functional magnetic resonance neuroimages. Comput Biomed Res.

[30] Beckmann CF, Smith SM (2004). Probabilistic independent component analysis for functional magnetic resonance imaging. IEEE Trans Med Imag.

[31] Jenkins M, Beckmann C, Behrens T, Woolrich M, Smith S (2012). FSL. NeuroImage.

[32] McKeown MJ (2000). Detection of consistently task-related activations in fMRI data with hybrid independent component analysis. NeuroImage.

[33] Schmithorst VJ, Holland SK (2004). Comparison of three methods for generating group statistical inferences from independent component analysis of functional magnetic resonance imaging data. J Magn Reson Imaging.

[34] Krafft CE, Schwarz NF, Chi L, Li Q, Schaeffer DJ, Rodriguez AL, Pierce JE, Dyckman KA, McDowell JE. The location and function of parietal cortex supporting reflexive and complex saccades, a meta-analysis of a decade of functional MRI data.

[35] Jaeggi SM, Seewer R, Nirkko AC, Eckstein D, Schroth G, Groner R, Gutbrod K (2003). Does excessive memory load attenuate activation in the prefrontal cortex? Load-dependent processing in single and dual tasks: functional magnetic resonance imaging study. NeuroImage.

[36] Linden DE, Bittner RA, Muckli L, Waltz JA, Kriegeskorte N, Goebel R, Singer W, Munk MH (2003). Cortical capacity constraints for visual working memory: dissociation of fMRI load effects in a fronto-parietal network. Neuroimage.

[37] Marklund P, Fransson P, Cabeza R, Larsson A, Ingvar M, Nyberg L (2007). Unity and diversity of tonic and phasic executive control components in episodic and working memory. NeuroImage.

[38] Miller EK, Cohen JD (2001). An integrative theory of prefrontal cortex function. Annu Rev Neurosci.

[39] Barch DM, Carter CS, Braver TS, Sabb FW, MacDonald A, Noll DC, Cohen JD (2001). Selective deficits in prefrontal cortex function in medication-naive patients with schizophrenia. Arch Gen Psychiatry.

[40] Cannon TD, Glahn DC, Kim J, Van Erp TG, Karlsgodt K, Cohen MS, Nuechterlein KH, Bava S, Shirinyan D (2005). Dorsolateral prefrontal cortex activity during maintenance and manipulation of information in working memory in patients with schizophrenia. Arch Gen Psychiatry.

[41] Tan HY, Sust S, Buckholtz JW, Mattay VS, Meyer-Lindenberg A, Egan MF, Weinberger DR, Callicott JH (2006). Dysfunctional prefrontal regional specialization and compensation in schizophrenia. Am J Psychiatry.

[42] Barbalat G, Chambon V, Franck N, Koechlin E, Farrer C (2009). Organization of cognitive control within the lateral prefrontal cortex in schizophrenia. Arch Gen Psychiatry.

[43] Jansma J, Ramsey N, Van Der Wee N, Kahn R (2004). Working memory capacity in schizophrenia: a parametric fMRI study. Schizophr Res.

[44] Manoach DS, Lindgren KA, Cherkasova MV, Goff DC, Halpern EF, Intriligator J, Barton JJS (2002). Schizophrenic subjects show deficient inhibition but intact task switching on saccadic tasks. Biol Psychiatry.

[45] Franke C, Reuter B, Schulz L, Kathmann N (2007). Schizophrenia patients show impaired response switching in saccade tasks. Biol Psychol.

[46] Greenzang C, Manoach DS, Goff DC, Barton JJ (2007). Task-switching in schizophrenia: active switching costs and passive carry-over effects in an antisaccade paradigm. Exp Brain Res.

[47] Petersen SE, Dubis JW (2012). The mixed block/event-related design. Neuroimage.

[48] Altmann EM, Gray WD. An integrated model of set shifting and maintenance. In: Proceedings of the third international conference on cognitive modeling. 2000; 2000: 17–24.

[49] Kieffaber PD, Kappenman ES, Bodkins M, Shekhar A, O’Donnell BF, Hetrick WP (2006). Switch and maintenance of task set in schizophrenia. Schizophr Res.

[50] Kieffaber PD, O’Donnell BF, Shekhar A, Hetrick WP (2007). Event related brain potential evidence for preserved attentional set switching in schizophrenia. Schizophr Res.

[51] Meiran N, Levine J, Meiran N, Henik A (2000). Task set switching in schizophrenia. Neuropsychology.

[52] Fassbender C, Murphy K, Foxe J, Wylie G, Javitt D, Robertson I, Garavan H (2004). A topography of executive functions revealed by functional magnetic resonance imaging..

[53] Broerse A, Crawford TJ, den Boer JA (2001). Parsing cognition in schizophrenia using saccadic eye movements: a selective overview. Neuropsychologia.

[54] Karch S, Leicht G, Giegling I, Lutz J, Kunz J, Buselmeier M, Hey P, Spörl A, Jäger L, Meindl T (2009). Inefficient neural activity in patients with schizophrenia and nonpsychotic relatives of schizophrenic patients: evidence from a working memory task. J Psychiatr Res.

[55] Holmes AJ, MacDonald Iii A, Carter CS, Barch DM, Andrew Stenger V, Cohen JD (2005). Prefrontal functioning during context processing in schizophrenia and major depression: an event-related fMRI study. Schizophr Res.

[56] Krasnow B, Tamm L, Greicius MD, Yang TT, Glover GH, Reiss AL, Menon V (2003). Comparison of fMRI activation at 3 and 1.5 T during perceptual, cognitive, and affective processing. NeuroImage.

