# Supplementary material for: Brain activation differences in schizophrenia during context-dependent processing of saccade tasks
Source: Behav Brain Funct. 2016 Jun 24;12:19. doi: 10.1186/s12993-016-0103-2 (PMC4919833; doi:10.1186/s12993-016-0103-2)

Effect of Trial Position on Saccade Performance Measures

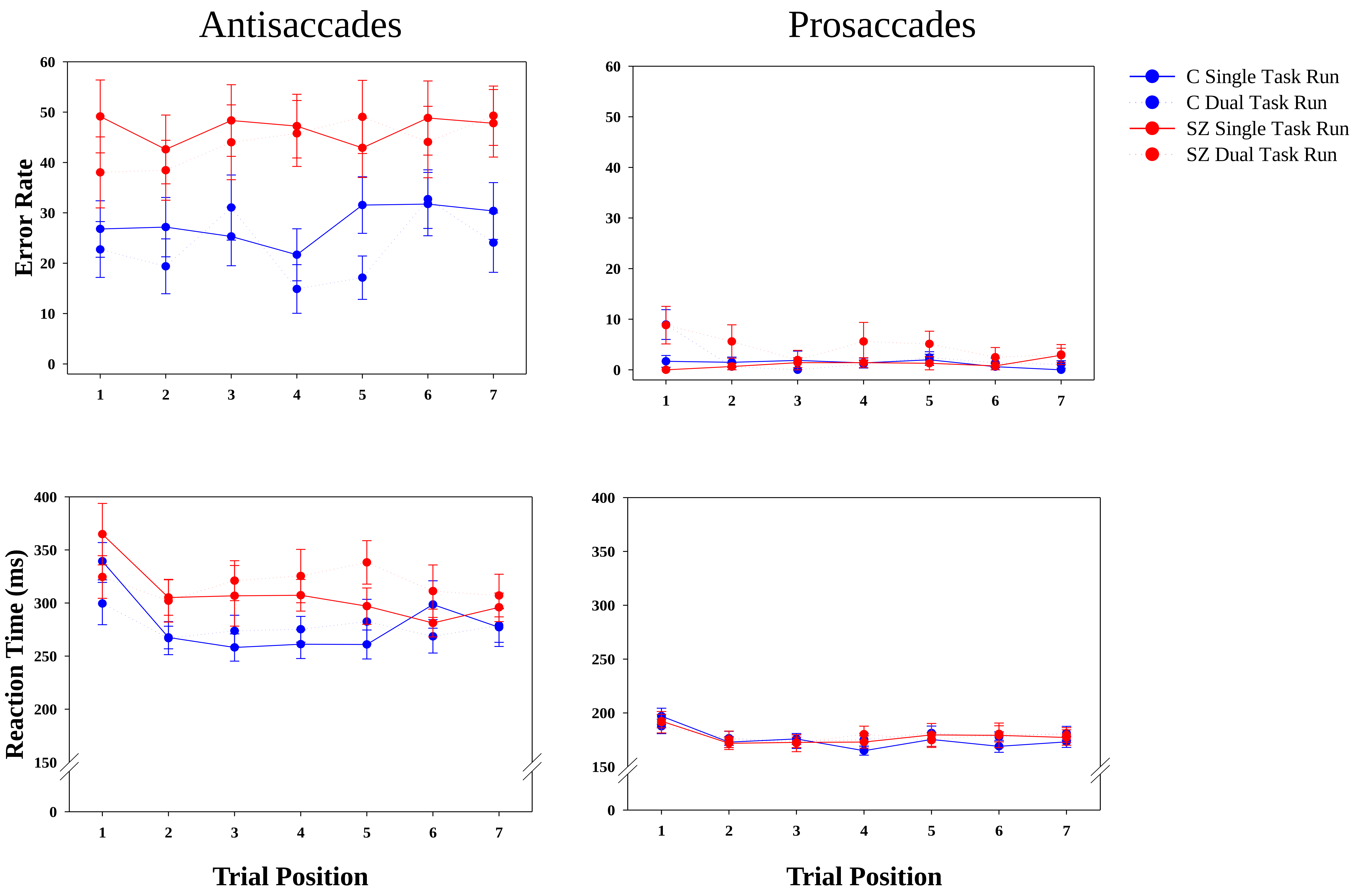

Supplement: Supplementary file 1 — 10.1186/s12993-016-0103-2 Trial Position and Performance Costs. Graphs show mean performance measures (SE) for antisaccades (left) and prosaccades (right) in each context plotted by trial position within a block. The schizophrenia group did not differ from the comparison group in either switch costs or mixing costs (similar pattern of means between the solid line and hashed line within each group). These patterns make it unlikely that differential switch or mixing costs between the two groups are responsible for the reported group-wise neural differences. C = comparison group, SZ = schizophrenia group. [file 12993_2016_103_MOESM1_ESM.pdf]
